# Psychometric properties and normative data of the childhood trauma questionnaire-short form in Chinese adolescents

**DOI:** 10.3389/fpsyg.2023.1130683

**Published:** 2023-02-27

**Authors:** Chang Peng, Junhan Cheng, Fajuan Rong, Yan Wang, Yizhen Yu

**Affiliations:** Department of Maternal, Child and Adolescent Health, School of Public Health, Tongji Medical College, Huazhong University of Science and Technology, Wuhan, China

**Keywords:** CTQ-SF, reliability, validity, norm, childhood maltreatment

## Abstract

**Background:**

The Childhood Trauma Questionnaire-Short Form (CTQ-SF) is a widely utilized instrument of childhood maltreatment (CM). However, psychometric properties and normative data of the CTQ-SF for Chinese adolescents are still unknown.

**Objective:**

To examine psychometric properties and normative data of Chinese version CTQ-SF in a nationally representative sample of Chinese adolescents, including internal consistency reliability, test–retest reliability, structural validity, and convergent validity.

**Method:**

A total of 20,951 adolescents aged 12 to 18 years were recruited from five provinces across China. Item analysis was used for 25 clinical items of the CTQ-SF. Confirmatory factor analysis was performed to examine fit indices of the factor structure. The Adverse Childhood Experiences Scale (ACEs) was used to evaluate convergent validity. The percentile ranks for scores of the CTQ-SF and each subscales were presented.

**Results:**

According to the results of three methods in Item analysis, Item 4 should be dropped. The remaining 24 clinical items achieved satisfactory fits in an alternative four-factor model. The alternative CTQ-SF showed acceptable internal consistency and the Cronbach’s α of the four subscales was 0.824 (Neglect), 0.755 (Sexual Abuse), 0.713 (Physical Abuse), and 0.666 (Emotional Abuse), respectively. Besides, test–retest reliability and convergent validity of the alternative CTQ-SF were also acceptable.

**Conclusion:**

The alternative four-factor model CTQ-SF exhibits good reliability and validity among Chinese adolescents. Additionally, the normative information of the CTQ-SF could provide practical support for determining severity of different subtypes of CM.

## Introduction

Childhood maltreatment (CM) is a major public health concern worldwide (WHO, 2020). Individuals who experience maltreatment during childhood and adolescence are more likely to develop various mental and psychological problems (Hughes et al., 2017), such as depression (Dunn et al., 2013), anxiety (Li et al., 2016), sleep disorders (Kajeepeta et al., 2015; Xiao et al., 2020), post-traumatic stress disorder (PTSD) (Messman and Bhuptani, 2017), bipolar disorder (Li et al., 2014a), personality disorder (Li et al., 2014b), substance use (Zhang S. et al., 2020), and suicidal behaviors (Grattan et al., 2019). More importantly, most of these adverse effects on mental health will last till to adulthood (Norman et al., 2012). Therefore, it has great significance to make efforts to prevent and address CM worldwide because of its high prevalence and direct or indirect damage to individuals and society (WHO, 2020). For targeted detection and intervention, the most primary and important task is to validate a suitable and efficient measurement tool to screen victims of CM.

A recent systematic review of 52 eligible self-reported measurements for CM found that the Childhood Trauma Questionnaire (CTQ) is the only scale that has the strongest psychometric properties and meets most standards of adequate reliability and validity (Saini et al., 2019). The CTQ is a 70-item self-administered inventory of abuse and neglect experiences, which was developed by Bernstein et al. (1994). In 2003, they revised the original CTQ and developed the Childhood Trauma Questionnaire-Short Form (CTQ-SF) (Bernstein et al., 2003), which showed better psychometric properties than the original CTQ (Saini et al., 2019). Thus, in this decades, the CTQ-SF is more widely used than the original CTQ in numerous studies internationally (He et al., 2019; Kongerslev et al., 2019; Aloba et al., 2020; Petrikova et al., 2021). In China, although some researchers have examined psychometric properties of the CTQ-SF among Chinese high school students, these previous studies only included small-size samples of adolescents with a convenience sampling method (Zhao et al., 2005; Zhang, 2011). As a consequence, these existing findings could hard to generalize for whole Chinese adolescents (He et al., 2019). Thus, the main purpose of the current study was to examine psychometric properties of the CTQ-SF among Chinese adolescents based on a large-size and representative sample across China.

The CTQ-SF has 28 items, including 25 clinical items (maltreatment evaluation items) and 3 validity items. The 25 clinical items are used to measure the five subtypes of CM: physical abuse, emotional abuse, sexual abuse, physical neglect, and emotional neglect (Bernstein et al., 2003). With regard to internal consistency, many previous studies have found that the physical neglect subscale generally has the poorest internal consistency among all five subscales (He et al., 2019; Aloba et al., 2020; Petrikova et al., 2021; Wu et al., 2022). In the light of these results, more research should try to re-examine or modify the items from the original CTQ-SF, especially for the physical neglect subscale (Georgieva et al., 2021). Besides, due to the cultural and social development differences between different countries and regions, it is not clear whether all items from the original CTQ-SF are suitable in the Chinese population (Charak et al., 2017; Rodriguez et al., 2019). Therefore, it is necessary to retest and screen items from the original CTQ-SF.

Bernstein et al. used confirmatory factor analysis (CFA) to confirm the five-factor model for 25 clinical items and demonstrated that the model had a good fit across several different population (Bernstein et al., 2003). However, some subsequent studies found that the original five-factor model of the CTQ-SF was not universal for clinical or community samples of adolescents and adults (Garrusi and Nakhaee, 2009; Klinitzke et al., 2012; Grassi-Oliveira et al., 2014). Because these previous studies have failed to replicate the original five-factor model (Gerdner and Allgulander, 2009; Kongerslev et al., 2019; Aloba et al., 2020). Meanwhile, other studies recommend that a four-factor structure of the CTQ-SF could be a good alternative, where items from the Physical neglect and Emotional neglect subscale were collapsed into one single Neglect subscale (Sacchi et al., 2018; Kongerslev et al., 2019; Şar et al., 2021). Some existing evidence supported that the conception of physical neglect and emotional neglect in the original CTQ-SF model was too interwoven to distinguish between these two forms of childhood neglect (Kim et al., 2011). However, to date, there is no empirical study to examine fit indices of the four-factor model. Therefore, the current study aims to determine whether the alternative four-factor model of the CTQ-SF has good structural fits in Chinese adolescents.

Beyond to the raw scores of the CTQ-SF and its subscales, normative data and cut-off scores can be applied to describe histories of abuse and neglect as well as its severity (Bernstein et al., 2003). Bernstein et al. first developed the norm of the CTQ-SF based on 286 patients with alcohol and drug abuse (Bernstein et al., 1994). In addition, Scher et al. represented another normative data of the CTQ-SF by recruiting 1,007 community residents between the ages of 18 and 65 years in the United States (Scher et al., 2001). This study reported scores of the CTQ-SF and its subscales based on percentiles *P*_25_, *P*_50_, *P*_75_, *P*_90_, and *P*_95_. However, although the above criteria to classify CM are widely used (Bernstein et al., 2003), there are still differences of cut-off values in various research (Jiang et al., 2018; Zhang S. et al., 2020). More importantly, all existing norm data and cut-off values of the CTQ-SF were developed *via* small-size samples from western countries. To date, it is still unknown whether these data are useful and suitable for Chinese adolescents. To the best of our knowledge, there is no published cut-off values or normative information of the CTQ-SF for a representative sample of Chinese adolescents.

To fill the gaps, this study aims to examine the psychometric properties and normative data of the Chinese version CTQ-SF based on a large-size and representative sample of Chinese adolescents. Our objectives are fourfold: First, we retest and screen items from the original CTQ-SF to determine whether all 25 clinical items are suitable and available in the context of Chinese culture. Second, we further examine the fit indices of the four-factor model of the CTQ-SF *via* confirmatory factor analyses (CFA). Third, we assess the internal consistency reliability, test–retest reliability, structural validity, and convergent validity of the CTQ-SF. Fourth, we aim to present the means, standard deviations, and percentile ranks of scores for the CTQ-SF and each subscale based on a nationwide representative sample.

## Materials and methods

### Procedure and participants

A multi-stage cluster sampling method was adopted from April to December in 2021. In Stage 1, China was divided into five main geographic locations (eastern, southern, western, northern, and central regions). Five representative provinces (Jiangsu, Guangdong, Yunnan, Gansu, and Hubei) were randomly selected from each region. In Stage 2, two cities were chosen randomly in each selected province. In Stage 3, we selected one district in an urban area and one county in a rural area from each selected city. In Stage 4, one junior high school and one senior high school were selected randomly in each sample district or county. In Stage 5, we used random digits to choose four or six classes from every grade (7th to 12th) in each selected school based on enrollment size. Finally, we invited all students in the selected class to participate in this survey voluntarily.

Among 23,207 questionnaires in total, 1,425 were excluded due to the missing data was more than 15% of items in the whole questionnaire, and 831 were excluded since their age was more than 18 or less than 12 years old. Finally, 20,951 students’ questionnaires were qualified for the final analysis, and the actual response rate was 90.28% (20,951/23,207). Besides, for assessing test–retest reliability, we recruited 1,500 of 23,207 participants to re-finish the questionnaire again after 6 months. Finally, 1,389 retest questionnaires were qualified, and the response rate was 92.60% (1,389/1500).

### Instruments

#### Childhood trauma questionnaire-short form

The original CTQ-SF was developed by Bernstein et al. (2003), and it was first translated into the Chinese version by Zhao et al. (2005). The CTQ-SF contained five subscales to evaluate five subtypes of CM. Each subscale was consisted of 5 items and each item was rated on a 5-point Likert scale (1 = Never true, 2 = Rare true, 3 = Sometimes true, 4 = Often true, 5 = Always true). Therefore, the score of each subscale ranged from 5 to 25. The total score of the CTQ-SF ranged from 25 to 125. In addition to 25 clinical items, the Minimization and Denial Scale (MD) was consisted of 3 items (Item 10, 16, and 22), which were not classified in any subtypes of abuse or neglect. Since the MD scale was used to reveal the denial of problems in CM, we did not process the MD scale in our study (Aloba et al., 2020; Petrikova et al., 2021).

#### Adverse childhood experience scale

The Adverse Childhood Experience Scale (ACEs) was also used to assess experiences of CM in prior to the age of 18 (Felitti et al., 1998; Finkelhor, 2018). In the present study, the ACEs was used to assess the convergent validity of the CTQ-SF (Schmidt et al., 2020; Petrikova et al., 2021). We used five items from ACEs to represent five subtypes of CM. Each item was dichotomized (0 = No, 1 = Yes) and the total score of the ACEs ranged from 0 to 5 in the study. According to previous studies conducted among Chinese population, the ACEs has acceptable validity and reliability among Chinese students and could be generalized to evaluate adverse childhood experiences for Chinese children and adolescents (Wang et al., 2018; Zhang L. et al., 2020).

### Data analysis

All data were analyzed with the 26th version of SPSS and AMOS software (IBM Corp.) for Windows. The significance level was set at *p* < 0.05 (two-sided) for all statistical significance testing. Descriptive statistics (M ± SD) were used to depict the CTQ-SF and its subscale scores. An independent sample *t*-test was used to compare the significant difference in scores between males and females (He et al., 2019). In addition to the *p*-values, the Cohen’s *d* effect size coefficient was evaluated (Cohen, 1988), in which effect size was either small (*d* = 0.20), medium (*d* = 0.50), or large (*d* = 0.80).

After excluded the 3 minimization/denial items (Item 10, 16, and 22), we conducted three methods of Item analysis for 25 clinical items from the original CTQ-SF (Bernstein et al., 2003). First, the correlation coefficients between each item and its subscale were used. If the correlation coefficient was less than 0.30 (*r* < 0.30), the item should be deleted. Second, the contribution of items was measured by factor loading analysis. If the maximum factor loading of an item was less than 0.40 (< 0.40), the item should be deleted. Third, if the internal consistency of a subscale did not decrease but increased after an item was removed, it generally indicated that this item reduced the homogeneity of the subscale. Thus, the item should be deleted. Finally, if an item was recommended to be deleted by all three methods of Item analysis, we would drop it in the following analysis (DeVellis, 1991; Sun and Xu, 2014).

Psychometric properties were further explored. The internal consistency of the CTQ-SF was evaluated with the Cronbach’s alpha (*α*) coefficient. In general, the Cronbach’s *α* > 0.70 is considered acceptable but *α* > 0.60 is also used (DeVellis, 1991). Due to scores of the CTQ-SF and the ACEs were not normally distributed, Spearman’s rho correlations (*r*) were calculated for assessing test–retest reliability, concurrent validity, and convergent validity (Jiang et al., 2018). The effect sizes of correlation coefficients were based on the criteria developed by Cohen (1988), in which effect size was either small (*r* = 0.10), medium (*r* = 0.30), or large (*r* = 0.50).

Confirmatory Factor Analysis (CFA) was done with the AMOS using the Maximum Likelihood Estimation of the covariance matrix input method. Since the *χ^2^*/df ratio has a limitation of inappropriately rejection for a model because of its sensitivity to large sample sizes (Joereskog, 1993). Therefore, the following fit indices were used to evaluate the four-factor model of the CTQ-SF: the comparative fit index (CFI), the goodness of fit index (GFI), and the root mean square error of approximation (RMSEA) (Steiger, 1990). Generally, the criteria of fit indices were used to evaluate an acceptable model: CFI ≥ 0.85, GFI ≥ 0.90, and RMSEA ≤0.08 (Brown, 2006; Hu & Hu and Bentler, 1998; Marsh et al., 2004).

## Results

### Demographic characteristics of the participants

Among 20,951 participants, females (50.4%) were slightly more than males (49.6%). Their age ranged from 12 to 18 years old, and the mean (SD) of age was 15.27 (1.75). More information is depicted in Table 1.

**Table 1:** Demographic information of participants [n (%)].

Variables	Total	Male	Female
Province			
Jiangsu	3,735 (17.8)	1,786 (17.2)	1,949 (18.5)
Guangdong	4,480 (21.4)	2,354 (22.6)	2,126 (20.1)
Yunnan	4,260 (20.3)	1,717 (16.5)	2,543 (24.1)
Gansu	3,875 (18.5)	2,016 (19.4)	1,859 (17.6)
Hubei	4,601 (22.0)	2,523 (24.3)	2,078 (19.7)
Residence			
Urban	10,896 (52.0)	5,829 (56.1)	5,067 (48.0)
Rural	10,055 (48.0)	4,567 (43.9)	5,448 (52.0)
Grade			
7–9	10,526 (50.2)	5,428 (52.2)	5,098 (48.3)
10–12	10,425 (49.8)	4,968 (47.8)	5,457 (51.7)
Age (year)			
12	2,230 (10.6)	1,167 (11.2)	1,063 (10.1)
13	4,135 (19.7)	2,085 (20.1)	2,050 (19.4)
14	3,488 (16.6)	1,828 (17.6)	1,660 (15.7)
15	2,923 (14.0)	1,457 (14.0)	1,466 (13.9)
16	4,031 (19.2)	1,896 (18.2)	2,135 (20.2)
17	2,956 (14.1)	1,419 (13.6)	1,537 (14.6)
18	1,188 (5.7)	544 (5.2)	644 (6.1)
Total	20,951 (100.0)	10,396 (49.6)	10,555 (50.4)

### Item analysis for 25 clinical items from the original CTQ-SF

The correlation coefficient between Item 4 and the Physical neglect subscale was less than 0.30. The maximum factor loading of Item 4 and Item 17 was less than 0.40. The Cronbach’s α of the Physical neglect subscale was 0.491. After dropping Item 4, the Cronbach’s α was increased to 0.494 (Table 2). According to the results of the three methods, Item 4 (My parents were too drunk or high to take care of the family) should be deleted in the alternative CTQ-SF.

**Table 2:** Results of Item analysis for 25 clinical items from the original CTQ-SF.

Subscale	Item	Description	Spearman’s *r*	Factor loading	Cronbach’s *α*		
					Before deletion	After deletion	Change
Physical abuse					0.713		
	A9	Hit hard enough to see doctor	0.422	0.449		0.691	−0.022
	A11	Hit hard enough to leave bruises	0.558	0.557		0.612	−0.101
	A12	Punished with hard objects	0.847	0.555		0.660	−0.053
	A15	Was physically abused	0.547	0.532		0.650	−0.063
	A17	Hit badly enough to be noticed	0.485	**0.393**		0.702	−0.011
Emotional abuse					0.666		
	A3	Called names by family	0.638	0.435		0.610	−0.056
	A8	Parents wished was never born	0.586	0.520		0.599	−0.067
	A14	Family said hurtful things	0.814	0.559		0.560	−0.106
	A18	Felt hated by family	0.302	0.508		0.647	−0.019
	A25	Was emotionally abused	0.473	0.496		0.638	−0.028
Sexual abuse					0.755		
	A20	Was touched sexually	0.763	0.473		0.715	−0.040
	A21	Hurt if did not do something sexual	0.522	0.495		0.709	−0.046
	A23	Made to do sexual things	0.489	0.510		0.694	−0.061
	A24	Was molested	0.623	0.504		0.703	−0.052
	A27	Was sexually abused	0.429	0.418		0.733	−0.022
Physical neglect					0.491		
	A1	Not enough to eat	0.527	0.725		0.422	−0.069
	A2	Got taken care of	0.690	0.469		0.410	−0.081
	A4	Parents were drunk or high	**0.264**	**0.306**		**0.494**	**0.003**
	A6	Wore dirty clothes	0.559	0.749		0.434	−0.057
	A26	Got taken to doctor	0.675	0.541		0.396	−0.095
Emotional neglect					0.857		
	A5	Made to feel important	0.769	0.602		0.836	−0.021
	A7	Felt loved	0.782	0.618		0.829	−0.028
	A13	Was looked out for	0.804	0.642		0.819	−0.038
	A19	Family felt close	0.811	0.635		0.829	−0.028
	A28	Family was source of strength	0.765	0.667		0.824	−0.033

Bold number refers to meet the deletion criterion.

### Goodness of Fit indices for the alternative CTQ-SF

**Figure 1:**
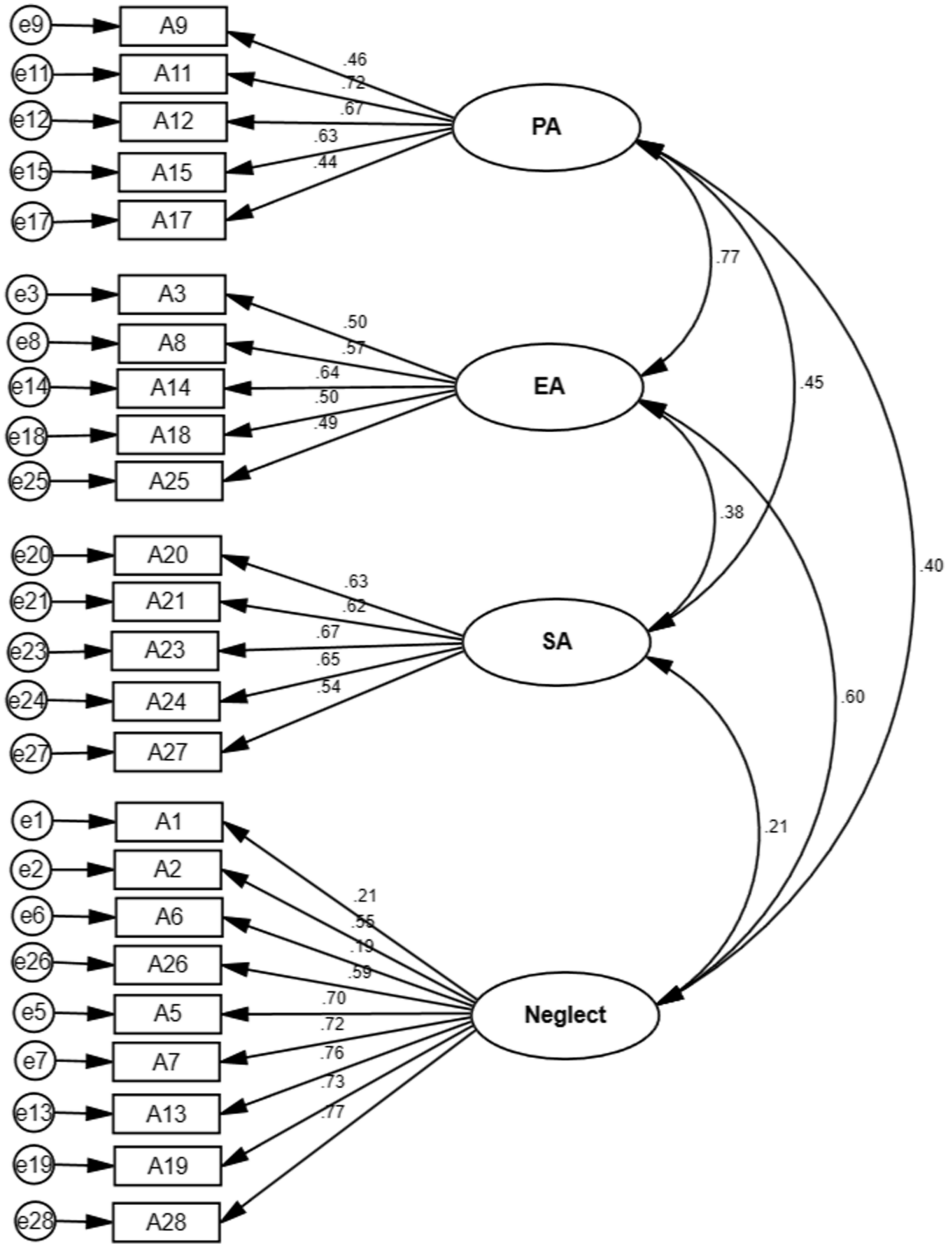
CFA path diagram of the alternative CTQ-SF model. PA: Physical Abuse; EA: Emotional Abuse; SA: Sexual Abuse.

The 9 items from the Physical neglect subscale (Item 1, 2, 6, and 26) and the Emotional neglect subscale (Item 5, 7, 13, 19, and 28) of the original CTQ-SF were collapsed into the Neglect subscale of the alternative CTQ-SF. The fit parameters of this four-factor model were also acceptable: *χ^2^/df* = 53.262, CFI = 0.908, GFI = 0.946, and RMSEA = 0.050 (Figure 1).

### Reliability and validity of the CTQ-SF

The Cronbach’s α of the original CTQ-SF was 0.852, and the Cronbach’s α for the five subscales from high to low was 0.857 (Emotional neglect), 0.755 (Sexual abuse), 0.713 (Physical abuse), 0.666 (Emotional abuse), and 0.491 (Physical neglect), respectively. The Cronbach’s α of the alternative CTQ-SF was 0.851, and the Cronbach’s α of the Neglect subscale was 0.824 (Table 3). The 6-month test–retest reliability coefficient of the alternative CTQ-SF was 0.548, and the corresponding correlation coefficients for the four subscales ranged from 0.256 (Physical abuse) to 0.509 (Emotional abuse). All these correlations were statistically significant (*p* < 0.01) (Table 3). The alternative CTQ-SF score was significantly correlated with the ACEs score (r = 0.355, *p* < 0.01). Besides, all the four subscales of the alternative CTQ-SF had significantly positive correlations with the ACEs score (*p* < 0.01), and the effect sizes of these correlations ranged from 0.195 to 0.379. The correlation coefficient between the original and alternative CTQ-SF was 0.999 (*p* < 0.01). The correlation coefficients between the Neglect subscale from the alternative CTQ-SF and the Physical neglect and Emotional neglect subscale from the original CTQ-SF were 0.829 and 0.910, respectively (*p* < 0.01) (Table 4).

**Table 3:** Reliability and validity of the alternative CTQ-SF.

Scale	Cronbach’s α		Test–retest reliability	Convergent validity
	Original	Alternative		
CTQ-SF	0.852	0.851	0.548^**^	0.355^**^
Physical abuse	0.713	0.713	0.256^**^	0.290^**^
Emotional abuse	0.666	0.666	0.509^**^	0.379^**^
Sexual abuse	0.755	0.755	0.308^**^	0.195^**^
Physical neglect	0.491	-	-	-
Emotional neglect	0.857	-	-	-
Neglect	-	0.824	0.501^**^	0.292^**^

^**^p < 0.01.

**Table 4:** Correlations between the original and alternative CTQ-SF.

	Original CTQ-SF	Physical abuse	Emotional abuse	Sexual abuse	Physical neglect	Emotional neglect
Alternative CTQ-SF	0.999^**^	0.499^**^	0.652^**^	0.302^**^	0.768^**^	0.866^**^
Physical abuse	0.499^**^	-	0.465^**^	0.234^**^	0.261^**^	0.327^**^
Emotional abuse	0.652^**^	0.465^**^	-	0.243^**^	0.330^**^	0.448^**^
Sexual abuse	0.302^**^	0.234^**^	0.243^**^	-	0.165^**^	0.169^**^
Neglect	0.938^**^	0.333^**^	0.445^**^	0.186^**^	0.829^**^	0.910^**^

^**^p < 0.01.

### Scores of the alternative CTQ-SF and the normative data

The mean scores of the alternative CTQ-SF are provided in Table 5. Males scored significantly higher than females in the CTQ-SF (*t* = 2.584, *p* = 0.010, Cohen’s *d* = 0.04), Physical abuse (*t* = 7.534, *p* < 0.001, Cohen’s *d* = 0.10), Sexual abuse (*t* = 10.953, *p* < 0.001, Cohen’s *d* = 0.15), and Neglect (*t* = 4.181, *p* < 0.001, Cohen’s *d* = 0.06). The score of the Emotional abuse subscale among females was significantly higher than males (*t* = 12.845, *p* < 0.001, Cohen’s *d* = 0.18). Due to the small effect sizes of sex difference for scores of the CTQ-SF and subscales (all Cohen’s *d* < 0.2), we computed normative scores in all participants regardless of different sex (Table 6).

**Table 5:** Scores of the alternative CTQ-SF and the four subscales by sex (Mean ± SD).

Scale	Total	Male	Female	*t*	*p*	Cohen’s *d*
CTQ-SF	33.17 ± 9.55	33.35 ± 9.78	33.01 ± 9.33	2.584	0.010	0.0356
Physical abuse	5.71 ± 1.82	5.81 ± 2.00	5.62 ± 1.62	7.534	<0.001	0.1044
Emotional abuse	6.72 ± 2.55	6.49 ± 2.41	6.94 ± 2.67	12.845	<0.001	0.1769
Sexual abuse	5.33 ± 1.47	5.44 ± 1.77	5.22 ± 1.10	10.953	<0.001	0.1493
Neglect	15.41 ± 6.57	15.60 ± 6.71	15.22 ± 6.41	4.181	<0.001	0.0579

**Table 6:** Normative data of the alternative CTQ-SF.

Scale	*P*_25_	*P*_50_	*P*_75_	*P*_90_	*P*_95_	*P*_97_
CTQ-SF	26	31	37	46	52	56
Physical abuse	5	5	5	7	9	10
Emotional abuse	5	6	8	10	12	13
Sexual abuse	5	5	5	6	7	9
Neglect	10	13	19	25	29	32

## Discussion

This is the first study to explore the psychometric performance and normative information of the CTQ-SF in a nationally representative sample of Chinese adolescents. Our study provides several major and new findings. First, one item (Item 4) has very low correlation and homogeneity with the original CTQ-SF in our data, which should be dropped in the alternative CTQ-SF. Second, when combining the Physical neglect and Emotional neglect subscale into the Neglect subscale, the alternative CTQ-SF with four subscales has better internal consistency than the original CTQ-SF with five subscales. Third, the first Chinese norm of the CTQ-SF will help to classify different severity of abuse and neglect among Chinese adolescents. These findings can largely benefit for promotion of the CTQ-SF in Chinese culture and society. Further, it can help scholars, clinicians, and social workers to detect and screen childhood maltreatment among Chinese adolescents.

According to the results of Item analysis in the current study, Item 4 (My parents were too drunk or high to take care of the family) is supposed to be removed because it is less related to the CTQ-SF and its subscale. Moreover, Item 4 can increase the heterogeneity of the Physical neglect subscale. Although this finding is consistent with the original CTQ-SF from Bernstein and his colleagues (Bernstein et al., 2003), some scholars support that not all items from the original CTQ-SF are appropriate for populations with different languages and cultures (Charak et al., 2017; Rodriguez et al., 2019; Şar et al., 2021). In many western countries, parents fail to take good care of their children for any reason, which is a typical behavior of neglect and is even subject to legal sanctions. However, under the traditional Chinese family values, if parents are unable to take care of their children, these children will usually be taken care of by their grandparents or other relatives. Therefore, this common phenomenon is not seen as physical neglect for most Chinese people. Additionally, compared to most western developed countries, Chinese laws and rules are more strict in regulating alcohol, drug use, or gambling. Consequently, the incidence of alcohol abuse, drug abuse, gambling, and other illegal acts in China is far lower than that in the western countries (Chen et al., 2019; Hoggatt et al., 2021). Therefore, it is rare that parents are too drunk or high to take care of their family in the Chinese community.

The Cronbach’s α of the original CTQ-SF reached accepted standards (α > 0.70), which reflects good internal consistency of the scale (He et al., 2019). However, the Physical neglect subscale has very low internal consistency coefficient (*α* = 0.491), which is much lower than an acceptable standard. This finding is consistent with most previous studies (Grassi-Oliveira et al., 2014; He et al., 2019; Aloba et al., 2020; Petrikova et al., 2021; Wu et al., 2022). The lack of homogeneity in the Physical neglect subscale may indicate a construction problem in the original CTQ-SF. The Physical neglect subscale contains three items focused on poverty and other two items focused on a lack of care. However, these behaviors refer to physical neglect can also represent to a lack of emotional care (English et al., 2005). Additionally, Gerdner et al. suggest that the poor internal consistency may be due to poor differentiation of physical neglect from emotional neglect (Gerdner and Allgulander, 2009). Moreover, these two seemingly separate factors are conceptually intermingled in the construct of neglect (Hernandez et al., 2013). Collectively, we can assume that the poor internal consistency of the Physical neglect subscale is not attributed to the Chinese translation version, but probably reflects a heterogeneous problem of the Physical neglect subscale in the original CTQ-SF (Petrikova et al., 2021).

After merging items of the Physical neglect and Emotional neglect subscale into the Neglect subscale, the four-factor model of the alternative CTQ-SF has better internal consistency than the original five-factor model. This is a surprising finding based on an innovative approach, which also partly cover up the heterogeneity of the Physical neglect subscale. In the alternative CTQ-SF, the Cronbach’s α for the Neglect subscale was high (*α* = 0.824). In addition, the correlations between the Neglect subscale of the alternative CTQ-SF and the Physical neglect and Emotional neglect subscale of the original CTQ-SF were very strong (r > 0.80). These results suggest a robust association between physical neglect and emotional neglect, and these two forms of maltreatment overlap to a large extend (Kim et al., 2011). In theory, to operationally distinguish the construct of neglect is difficult because nearly all definitions are based on personal discernment of lack of care (English et al., 2005). What’s more, neglect can generally occur emotionally even when needs are met physically (Grassi-Oliveira et al., 2014).

The normative data of the CTQ-SF among Chinese adolescents in this study are quite different from those from the previous study (Scher et al., 2001). For example, the percentiles of *P*_25_, *P*_50_, *P*_75_, and *P*_90_ of the PA subscale in the American community population are 5, 6, 7, and 9, respectively (Scher et al., 2001). In our study, the numbers are 5, 5, 5, and 7, respectively. Moreover, the *P*_25_, *P*_50_, and *P*_75_ of the EA subscale in American adults are 5, 5, and 7, respectively. Among Chinese adolescents, the *P*_25_, *P*_50_, and *P*_75_ are 5, 6, and 8, respectively. The results indicate that the score distribution of the CTQ-SF varies greatly between different populations, so the norm of the CTQ-SF may also change greatly. Therefore, the development of norms should take account of the social and cultural differences between different countries. Besides, the cut-off values of the CTQ-SF should be modified based on specific normative data. If scholars use the same norm and cut-off values in different research populations, the prevalence of childhood maltreatment will be significantly overestimated or underestimated. For example, when we take the cut-off value (score ≥ 10) of the Physical abuse subscale according to Bernstein et al. (2003), the prevalence of physical abuse was near 3% for Chinese children and adolescents aged 12 to 18 in the current study. Apparently, the prevalence is much lower than that reported in a recent meta-analysis, which indicated that the prevalence of physical abuse in Chinese primary and secondary school students is 20% (95%CI: 13–27%) (Wang et al., 2020). Therefore, the formulation of the CTQ-SF norms and cut-off values should consider different characteristics of participants. On the other hand, the normative and psychometric data of the CTQ-SF from our report could largely help researchers and educators to screen the prevalence of childhood maltreatment.

## Limitations

The current study has several limitations. First, we recruited a large sample of Chinese adolescents from 40 junior and senior high schools. However, this study did not recruit community adolescents who are unenrolled in school. Although the proportion of these unenrolled adolescents aged between 12 and 18 is very small in China, we can also recruit some unenrolled adolescents in future research to make our participants more representative. Second, the test–retest reliability of the alternative CTQ-SF in this study was 0.548, which did not meet a common standard of more than 0.70. The result may attribute to the duration between the first survey and the retest survey is being 6 months, which is far longer than a common duration of 2 to 4 weeks for the test–retest survey. In future research, we are supposed to attempt to overcome the impact of COVID-19 for field investigation and shorten the duration of test-retesting. Third, although we have developed the first norm of the CTQ-SF among Chinese adolescents in the current study, we are unable to dichotomize the experience of abuse or neglect *via* valid cut-off values. In the next step, we will conduct a structured clinical interview for participants and depict the receiver operating characteristic curve (ROC) for the Chinese version CTQ-SF (Walker et al., 1999), which will have great significance for the diagnosis of childhood maltreatment.

## Conclusion

The findings from the current study support a good reliability and validity of the alternative CTQ-SF in Chinese adolescents, which includes 24 clinical items and four subscales: the Physical abuse, Emotional abuse, Sexual abuse, and Neglect. Moreover, the first Chinese norm of the CTQ-SF could provide great benefits for scholars, educators, and clinicians to detect and screen adolescents’ maltreatment experiences and reveal the epidemiological characteristics of abuse and neglect among Chinese adolescents.

## Data availability statement

The raw data supporting the conclusions of this article will be made available by the authors, without undue reservation.

## Ethics statement

The studies involving human participants were reviewed and approved by The Medical Ethics Committee of Tongji Medical College, Huazhong University of Science and Technology. Written informed consent to participate in this study was provided by the participants’ legal guardian/next of kin.

## Author contributions

CP developed the initial manuscript. JC and FR were responsible for the data collection and the data analysis. YW contributed substantially to the revision and refinement of the final manuscript. YY guided the overall design of the study. All authors contributed to the article and approved the submitted version.

## Funding

This work was supported by grants from the National Natural Science Foundation of China (grant numbers 81973066 and 82173541).

## Conflict of interest

The authors declare that the research was conducted in the absence of any commercial or financial relationships that could be construed as a potential conflict of interest.

## Publisher’s note

All claims expressed in this article are solely those of the authors and do not necessarily represent those of their affiliated organizations, or those of the publisher, the editors and the reviewers. Any product that may be evaluated in this article, or claim that may be made by its manufacturer, is not guaranteed or endorsed by the publisher.
